# Perceived benefit of yoga among adults who have practiced yoga for a long time: a qualitative study

**DOI:** 10.1186/s13030-023-00276-3

**Published:** 2023-05-15

**Authors:** Şengül Akdeniz, Özlem Kaştan

**Affiliations:** 1 Department of Healthcare Services, Vocational School of Healthcare Services, Akdeniz University, 07070 Antalya, Turkey; 2 Department of Medical Services and Techniques, Vocational School of Health Services, University, 07070 Antalya, Turkey

**Keywords:** Yoga, Perceived benefit, Mental health, Physical health, Qualitative study

## Abstract

**Background:**

Previous quantitative studies have shown many of the perceived benefits of yoga practice on the mind and body. Although there are many quantitative studies in the international literature on yoga, the number of qualitative studies showing the experience of yoga practice is insufficient. An accurate demonstration of yoga participents experiences, opinions, and judgments on this subject requires a qualitative, rather than a quantitative approach.

**Purpose:**

This study was to explore the benefit perceived by adults who have practiced yoga for a long time.

**Design and method:**

This qualitative study is based on a hermeneutic–phenomenological approach. The research sample consisted of 18 adults who volunteered to participate in the research and regularly joined yoga practice. The study data were collected through individual and focus group interviews with the participants who practise yoga and analyzed by content analysis method.

**Results:**

We created five themes. Themes coded by researchers: meaning of the concept of yoga (theme 1), physical, mental, and social state before starting yoga (theme 2), reasons for practising yoga (theme 3), the participants' experiences related to their physical and mental health and social relationships (theme 4), and difficulties of doing yoga (theme 5). In addition, individuals in the study reported their perceptions of the concept of "yoga" through the creation of metaphors that completed the following sentence “yoga is like ……”. These metaphors were used to give insight into the participants deep feelings about yoga.

**Conclusions:**

Both in their individual and focus group interviews, almost all of the participants described that doing yoga has positive benefits on the mind and body. The positive experiences of the participants in the study included decrease in pain and flexibility, increase in sleep quality, development of positive personality traits, increase in self-esteem, and coping with anxiety and stress more effectively. Because the study was qualitative and long-term, it was able to evaluate the beliefs, attitudes and behaviors of individuals in a realistic, systematic, and detailed manner.

## Background

While low stress levels create a positive impact, higher ones adversely affect the functioning of all organs and systems in the organism and may lead to various diseases. It is therefore important to manage to control stress and cope with it [1]. Various techniques are used to control or reduce stress and change stress-coping strategies and behaviors. Yoga is a technique for relaxing the mental health and controlling stress via internal and external resources [2]. Studies on yoga suggest that yoga is an effective method of coping with stress, leading to growing self-awareness and a positive increase in quality of life [3–7]. Studies have found that yoga has positive effects on coping with stress, increasing sleep quality, growing self-confidence, relaxation, and strengthening memory and concentration [8, 9]. A randomized controlled study on yoga puts forward that yoga is effective in coping with neuronal and endocrine changes and stress, and also decreases the prevalence of mental illnesses by reducing depression and anxiety [9]. The study conducted by Tulloch et al. (2018) shows that yoga has a significant role in improving balance, movement, and mental health [6].

Studies suggest that yoga has positive effects on physical health as well as on mental health. It is reported that the use of yoga-based exercises as an alternative complementary treatment has a positive effect on various chronic diseases, such as paralysis [10], arthritis [11], obesity [9], type 2 diabetes [12], cardiovascular disease [13], metabolic syndrome [14], and cancer [15]. There are many studies showing the positive physical, mental, and social effects of yoga. As yoga has spread all over the world and has been nourished by the cultures in the areas where it has spread, many types of yoga have emerged. The most important effects of all types of yoga are that they teach the power to reveal the positive qualities of human beings and to reduce their deficiencies [7–15].

Tulloch et al. suggest that yoga allows a person to focus, enter a peaceful state, and then incorporate the benefits of this relaxing experience into their lifestyle [6]. Yoga reveals the integrity of mind, body, muscle, bone, and spiritual in a holistic manner. When practicing yoga, people know which muscle is moving in each stretching movement, and a physical integrity is achieved by concentrating on it. Yoga is a great form of exercise focused on human anatomy for physical health. When it comes to yoga movements, many people may think of some acrobatic movements and difficult postures. Because each individual's anatomical structure, joints and bones are different, the limits of being able to do these postures are also different. As the movements are performed, the body opens and stretches. Yoga poses, which have hundreds of variations today, have been diversified over time by yoga experts according to their knowledge of anatomy and the areas they focus on. The perception of yoga as a part of physical culture has been attributed to its stress-reducing and relaxing properties based on anatomical and physiological assumptions [2, 4–6]. Yoga is also used as an alternative treatment for some diseases. According to the literature, there are no studies showing negative physical, mental, or social effects of yoga. However, more studies are needed on exactly which health problems yoga has a positive effect on.

Although yoga is known to be especially effective in protective and improving the health of healthy adults, scientific studies in this field are both few in number and are usually short-term studies. Among the studies that have been conducted on yoga, qualitative studies in which the subjective experiences of individuals practising yoga are questioned are limited. In the literature review, there was no qualitative study on yoga conducted in Turkey that take into account long-term lifestyle and for which positive effects are revealed. Yoga is less well known and practiced in Turkey compared to other countries. One of the first reasons for this is that yoga is not well known in Turkey. In addition, the income and education level compared to other countries constitute other factors affecting the promotion and demand of yoga education and philosophy. Yoga as an academic research subject in Turkey started to intensify after 2015. The exact number of yoga practitioners in Turkey is not known. Compared to abroad, there are very few yoga instructors and yoga centers in Turkey compared to other countries. It is thought that this study will contribute to the recognition of yoga in Turkey.

This study was done for a long period, two years, to investigate how the emotions, thoughts, and experiences of adults related to the effect of yoga-based exercises on their physical, mental, emotional, belief, and social health states.

## Methods

### Study design and sampling

In order to explore the benefit perceived on the physical, spiritual, emotional and social situations by adults who have practiced yoga for a long time, we used a qualitative research method that investigates the event(s) related to a situation in depth within a person’s own boundaries (environment, time, etc.) to do a holistic analysis [16]. The study used a holistic, multiple design; a case study design in which more than one situation perceived as holistic is handled and compared. The population of the study consisted of the individuals practising yoga.

In a qualitative study, data collection is terminated when the data reaches a saturation point, when there is no new information, and/or data starts to repeat itself [16]. Therefore, our research participants consisted of 18 individuals who volunteered to participate in the research and regularly joined yoga practice. Individual interviews were done in January 2020, and focus group interviews were held in February 2020. The participants were given information about the place and time of the meeting, and a meeting room was made available and arranged in a way that was suitable fort he study.

### Data collection

Data was collected through individual and focus group interviews with the participants and through participant observation. The participants practiced yoga two days a week. For this reason, focus group participants were divided into two groups of nine, with the groups meeting on different days, and the group participants were interviewed before and after doing yoga. The group discussions lasted approximately one hour. Focus group interviews allowed us to see social interactions before and after yoga.

The interviews were conducted via a semi-structured interview form. A Personal Information Form and a Semi-Structured Interview Form were used during the interviews. It took an average of 45–50 min. to conduct these interviews. The researcher acting as a participant observer went to a yoga studio selected for participant observation two days a week from April 5, 2018 to March 11, 2020. During this time, social communication was observed in different areas of the studio such as classrooms, changing rooms, and kitchen. Yoga classes are voluntarily offered at the yoga studio and there may be changes in the profile of the students who join these classes. The researcher attended yoga classes between 12.00–13.00 h, twice a week, for 2 years. During the first year, the participant observer researcher attended yoga classes almost without missing any of them, and recognized changes in his body and thinking about what yoga is and its effects. The researcher acting as a participant observer observed all participants individually from the beginning of his participation in the yoga classes. Participants did their physical activity of yoga on a mat that was suitable for the person's height, that was heat-insulated, and that was not too high and did not allow slipping. It was done under the guidance of a trainer in a yoga hall appropriate for yoga. The participants were observed to have experienced difficulties in performing some physical movements due to their age and flexibility characteristics, particularly in the initial phase. It was observed that as time passed and yoga activity became a habit among the participants, these difficulties decreased in parallel with the duration of yoga. Moreover, both the atmosphere of the yoga hall and the group interaction of all participants were observed.

#### Personal information form

The personal information form contains questions about seven items: gender, age, education, occupation, chronic disease, chronic pain, and duration of yoga practice.

#### Semi-structured interview guide

The semi-structured interview form contains six questions including the meaning of the concept of yoga, the physical, mental and social state before starting yoga, the reasons for practising yoga, the effects of yoga on the physical, mental and social state, the difficulties of doing yoga, and a metaphor to explain what yoga is like. In addition, the researcher asked probing questions in order to deepen and clarify the issue during the individual interview.

### Data analysis

The data obtained as a result of individual interviews was analyzed through the descriptive analysis method. This analysis method addresses the statements and results of the participants regarding the research question. Data is summarized and interpreted according to the pre-determined themes in this type of analysis [16]. Data analysis was done with the Nvivo 10 package program. Firstly, the data collected as a result of individual interviews and participant observations was documented, and upon completion of all individual interviews, themes were created. Researchers initially worked independently while creating themes, and then the themes were compared and common themes were formed. In the data analysis, the researchers obtained mostly consistent results. When the researchers did not agree with each other, the interview text was read in detail in order to obtain a common impression by making an in-depth naive reading. For the reliability of the research, two experts who did not know about the study were asked to code according to the themes. Apart from descriptive data, numerical analyzes of data collected as a result of individual interviews were also presented and interpreted in the study. Moreover, in order to increase the validity and reliability of the study, direct quotations were made from participant statements.

### Ethical aspect of research

Ethical approval from ……University Clinical Research Ethics Committee (approval number: 70904504/299, date of approval 05/07/2019) and permission from the yoga trainer who contributed to the research were obtained for carrying out the study. This study was conducted in accordance with the principles of the Helsinki Declaration. The yoga practitioners who participated in the study were informed that the research was a scientific study, that an individual interview would be held, and that their real names would not be used in data analysis, each participant was given a code number (P1–P18) and their informed voluntary consent was obtained.

## Results

Analysis of the Personal Information Form found that the age range of the 18 participants was between 23 and 68 years, 6 were between 40–50 years old and 16 were women. Considering the occupations, 4 were lecturers, 3 were civil servants, 6 were students, teachers, or retired teachers (2 each), and 1 each was a nurse, painter, administrative assistant, massage therapist, or housewife. With respect to the disease state of the participants, it was revealed that 15 of them had a history of chronic disease, 11 had chronic pain, and 3 had anxiety disorder. The duration of yoga practice varied from 2 to 5 years (Table 1).

**Table 1:** Sociodemographic data (*n* = 18)

Participant	Gender	Age	Education	Occupations	Chronic disease	Chronic pain	Duration of yoga practice (years)
P1	Female	23	High school	Student	Yes	No	2
P2	Female	68	University	Teacher	Yes	Yes	3
P3	Male	27	University	Student	No	No	2
P4	Female	59	University	Lecturer	Yes	Yes	3
P5	Female	50	University	Administrative assistant	Yes	No	4
P6	Male	39	University	Massage therapist	No	No	5
P7	Female	41	University	Civil servant	Yes	Yes	3
P8	Female	51	University	Painter	Yes	Yes	2
P9	Female	28	University	Lecturer	Yes	Yes	2
P10	Female	40	University	Teacher	Yes	Yes	2
P11	Female	35	University	Lecturer	No	No	2
P12	Female	45	University	Nurse	Yes	Yes	5
P13	Female	67	University	Retired teacher	Yes	Yes	2
P14	Female	64	University	Retired teacher	Yes	No	2
P15	Female	47	University	Lecturer	Yes	Yes	3
P16	Female	50	University	Civil servant	Yes	Yes	2
P17	Female	60	University	Civil servant	Yes	No	2
P18	Female	59	Primary school	Housewife	Yes	Yes	3

We created five themes. Themes coded by the researchers: meaning of the concept of yoga (theme 1), physical, mental and social state before starting yoga (theme 2), reasons for practising yoga (theme 3), the participants' experiences related to their physical and mental health, and social relationships (theme 4), and difficulties of doing yoga (theme 5). In addition, individuals in the study reported their perceptions of the concept of "yoga" through the creation of metaphors that completed the following sentence “yoga is like ……”. These metaphors were used to give insight into the participants deep feelings about yoga.

### Theme 1: Meaning of the concept of yoga

Of the participants, 11expressed the meaning of the concept of yoga with the theme “exercise”, while 5 replied “healthy life” and “calmness”, 3 ‘harmony’, and 2 "awareness" and “balance” (Table 2). Some of the participants' statements regarding the meaning of the concept of yoga are as follows:

- *For me, the concept of yoga means standing against the speed culture created by our current age, and the calmness that occurs as a result this stance (P3) *(Theme-calmness)*.*
- *Yoga means ensuring harmony between the inner world and the outer world for me (P12)* (Theme-harmony-balance).
- *It is a form of physical exercise that focuses on breathing and protects our physical and mental health through stretching movements (P4)* (Theme-exercise-healthy life).
- *It's the best time I spare to myself. I become aware of my whole body (P13)* (Theme-awareness).
- *It is the movements and rest that calm your mind and make you feel mentally fit while stretching and resting the body through relevant body movements (P9)* (Theme-exercise).

**Table 2:** Theme and sub-theme experiences of the participants who practice yoga

Themes/ *sub-themes	*n* = 18 (The number of participants)
**Theme 1. Meaning of the Concept of Yoga**	**18**
*Healthy life	**5**
*Exercise	**11**
*Awareness	**2**
*Balance	**2**
*Calmness	**5**
*Harmony	**3**
**Theme 2. Physical, Mental and Social State before Starting Yoga**	**18**
*Physical ailments	**3**
*Sedentary life	**3**
*Doing sports other than yoga	**6**
*Feeling negative emotions	**3**
*Fatigue	**4**
*Social isolation	**1**
*Coping difficulty	**4**
*Anxiety	**1**
**Theme 3. Reasons for Practising Yoga**	**18**
* Healthy life	**8**
* Reducing chronic pain	**9**
*Coping with stress	**1**
* Raising awareness	**6**
*Increasing positive thinking and belief	**1**
* Being calm	**3**
**Theme 4. Experiences of the Participants' Physical, Mental and Social State**	**18**
* Reducing chronic pain	**9**
*Increasing sleep quality	**4**
*Increasing positive personality traits	**5**
*Increasing self-esteem	**4**
*Raising awareness	**5**
*Increasing positive emotions	**12**
*Relieving	**11**
*Coping with stress	**2**
**Theme 5. Difficulties of Doing Yoga**	**18**
*Physical difficulty	**10**
*Mental difficulty	**1**

### Theme 2: Physical, mental, and social state before starting yoga

After the participants were asked about their physical, mental and social state before starting yoga, they gave the following responses: 6 were "doing sports other than yoga"; 4 were "fatigued" and had "coping difficulty"; 3 had " physical ailments', 'sedentary life' and felt 'negative emotions', and 1 had 'social isolation' (Table 2).

Some of the participants' statements about their physical, spiritual and social state before starting yoga are as follows:

- *I couldn't find time to rest, I often had neck pain, and I had lung disease in the winters *(P10) (Theme—physical ailments).
- *I had fatigue, leg pain and oedema, as well as varicose pain that did not go away* (P17) (Theme—physical ailments).
- *I had a monotonous life, I was a fairy godmother, I always said yes to everyone, and I did not have time to spare to myself and to be on my own (P18)* (Theme-sedentary life).
- *I was interested in sports that required more speed. I didn't know the importance of slowing down and awareness of body (P11)* (Theme-sports other than yoga).
- *I was in a stressful state with difficult-to-cope emotions* (P1) (Theme-feeling negative emotions- coping difficulty).
- *Before starting yoga, I was less tolerant of events and feeling more lonely, and thanks to yoga, I believe that I have overcome these negative emotions, and my presence in the universe is so pleasant and I am grateful for being a part of the universe (P5)* (Theme-feeling negative emotions).
- *Before I started doing Yoga, especially my mind was like a machine running full-time (P3)* (Theme-fatigue).
- *I had a very intense pace of work. Since my husband worked out of the city, it was really exhausting and wearing to assume the responsibility of two children, to be both a mother and father, to run a large hospital and to attend classes at school (P15)* (Theme-Coping difficulty).
- *I've been trying to do yoga with you for two years. During this period, I had angio ablation because of rhythm disturbance, but it was not successful and drug treatment was started. Meanwhile, I had anxiety and a fear that I didn't know about, I started yoga at this very moment, my fears decreased every day, I felt my soul healed, and after five months, I stopped taking antidepressants. I felt relief in my heart (P16)* (Theme-anxiety).

### Theme 3: Reasons for practising yoga

Participants stated that they practised yoga for a healthy life (8), to raise awareness (6), to reduce pain (9), to be calm (3), to cope with stress (1), and to increase positive thinking (1) (Table 2).

Some of the participants' statements regarding their reasons for practising yoga are as follows:

- *Yoga strengthens the immune system, allows you to have a healthy skeletal system, and even strong and flexible muscles, and it regulates the circulatory system, positively affects the digestive system, reduces stress, improves sleep quality, and improves communication skills and self-confidence (P6) *(Theme—healthy life).
- *When I do yoga, I feel that I think in a healthier way and my body is healthier. I also feel mentally and spiritually purified (P9)* (Theme—healthy life).
- *Severe cricks started in my back. I had crick in my back for five times in a year, I stayed in bed for months and had injections. The quality of my life decreased so much that I was afraid to even go for a walk. I went to a doctor and the doctor said I was having a heavy muscle spasm, and he recommended yoga. My brother had been practising yoga for about a year and he was so happy, I started it thanks to him. I was in a lot of pain on the first day when I started yoga. And when I got out of the yoga hall, a miracle happened. I felt so good, I can't describe it. I got healthy again (P8)* (Theme—Reducing pains).
- *I started yoga because I thought it would relax my mind. I thought it would help me relax and get me out of the stressful situation. And it helped as I thought (P15)* (Theme—coping with stress).
- *Being able to be on my own and integrating with myself mentally and physically, I feel my calm and peaceful mood in my daily life increased, and I feel my muscles relax and my body relax (P16)* (Theme- raising awareness).
- *Events that used to worry me for days and make me nervous now make me laugh. I can focus better now. And most importantly, my energy has gone up. Now I can easily get rid of the usual negativities around me (P10)* (Theme—increasing positive thinking).

### Theme 4: Experiences of the participants' physical, mental and social state

Participants stated the effects of yoga on their physical, mental and social states as follows: increasing positive emotions (12), relieving (11), reducing pain (9), increasing positive personality traits and raising awareness (5), increasing sleep quality and self-esteem (4).

Some of the participants' statements about the effects of yoga on their physical, mental and social states are as follows:

- *I had back and neck pain due to working at a desk and stress. I had less pain for 2 years (P10)* (Theme-reducing pain).
- *Physically, my muscle structure has been strengthened, and I can see it clearly. My spinal posture has improved. I feel it is good for my herniated disc. It relieved my back pain (P12)* (Theme- reducing pain).
- *My back pain has decreased, I have become more flexible and I feel more peaceful. (P4)* (Theme- reducing pain).
- *My back pain with which I had struggled before yoga is over… I have not taken a single muscle relaxant for 7 months, I have not had an injection… I had headaches like migraine and they decreased greatly (P8)* (Theme- reducing pain).
- Thanks to the breathing exercises, the quality of my sleeping pattern got better (P13) (Theme- increasing sleep quality).
- *My sleeping pattern has become regular by proper breathing since I started practicing yoga, (P17)* (Theme- increasing sleep quality).
- *Psychologically, yoga has transformed me into a more balanced and calm person (P1)* (Theme-increasing positive personality traits).
- *On the days when I practice yoga, my brain works harder, and I'm more humorous and tolerant, smiling and less forgetful (P2)* (Theme-increasing positive personality traits).
- *Yoga allowed me to become stronger psychologically, and it increased my quality of life, and since it motivated me positively, based on the belief that I could do whatever I wanted, positive thoughts in my soul were reflected on my body and created an integrity (P10)* (Theme- increasing self-esteem-increasing positive emotions).
- *Being aware of my body and emotions has been good for my physical and mental health (P11)* (Theme—increasing awareness).
- *For me, yoga takes you on a journey into your inner world. It raises awareness of your body, your inner world, and your essence. It teaches to be unified with the body, to listen to it and to perceive the messages coming from it (P12)* (Theme- raising awareness).
- *I feel much more energetic, I do not get sick, I do not feel pain in my body, I do not get tired, I do not get angry, I am calmer when communicating with people and I can understand them. Since I feel stronger, I have no difficulty in performing my work and I can work with pleasure (P6)* (Theme- increasing positive emotions).
- *I feel energetic thanks to the deep breath I take in yoga. The feeling of fatigue is over (P16)* (Theme—relieving).
- *I feel a relief in my joints, and I do not feel fatigue (P2)* (Theme- relieving).
- *But I think yoga decreases the stress level and increases the happiness hormone, and ensures spiritual, mental and physical balance (P16)* (Theme—coping with stress).
- *I can say that it allows me to control my anxiety because it calms my mind psychologically (P9)* (Theme—coping with stress) (Table 2).

### Theme 5: Difficulties of doing yoga

Participants described, their difficulties in practising yoga, with the themes of physical difficulty (10) and mental difficulty (1).

Some of the participants' statements about the difficulties of practicing yoga are as follows:

- *While practising yoga, you have to throw away the thoughts in our head for 1–1.5 h, and if you push yourself away for a moment, you will break from the group in yoga because the movements are coordinated with commands. This is of course difficult after a certain age (P2)* (Theme- mental difficulty). This participant (P2) is the oldest participant of the study group.
- *Some of the movements we do physically are difficult for me, which is because I didn't do yoga or any sports when I was a child, so it can sometimes be difficult for me to manage my muscles (P10)* (Theme—physical difficulty) (Table 2).

### Metaphors about doing yoga

**Fig. 1:**
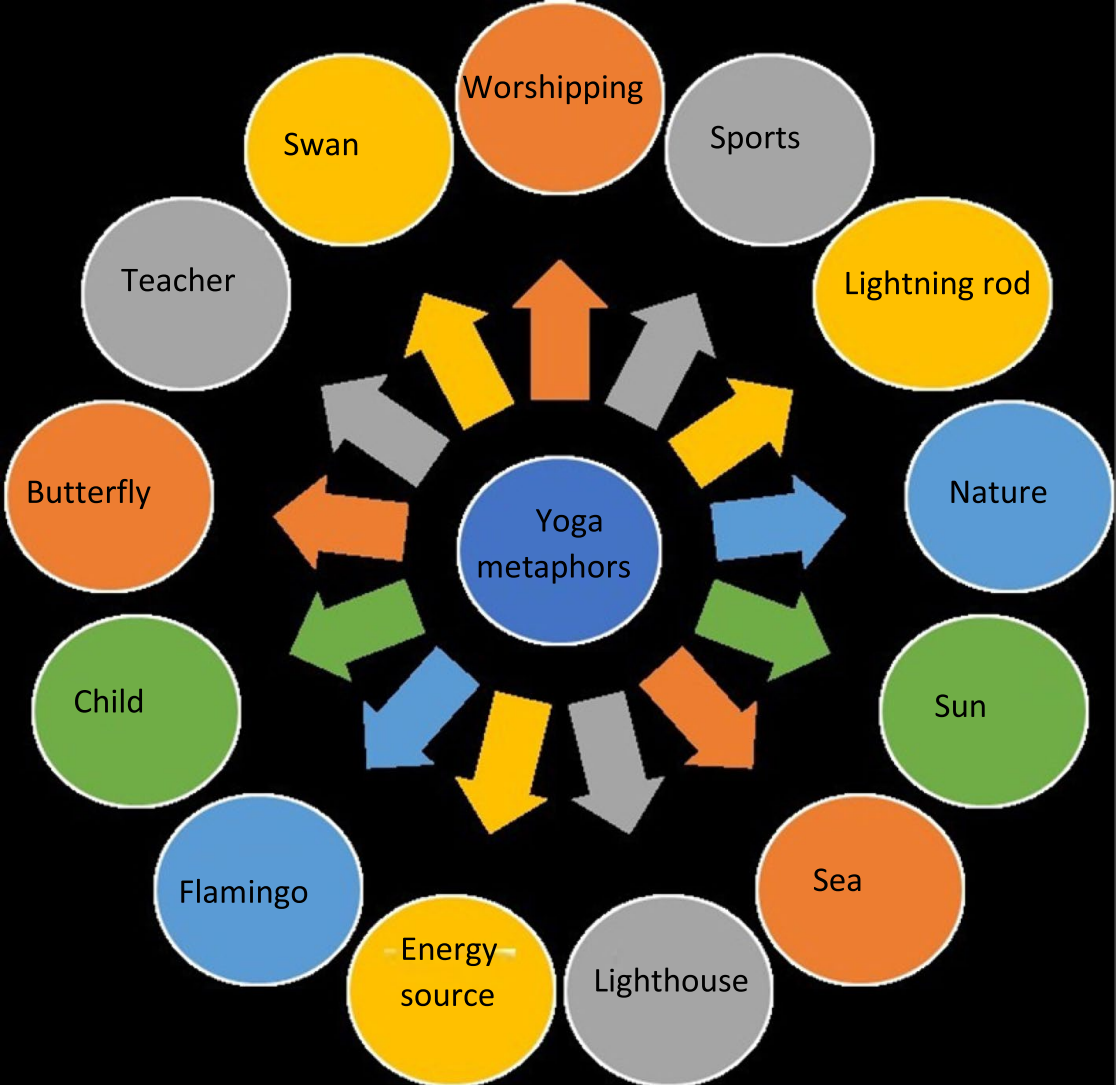
Participants’ metaphors for practising yoga

When participants were asked to compare their experience of doing yoga to something thirteen metaphors emerged (Fig. 1). The responses to the question about the metaphor for practicing yoga were generally collected within the framework of supporting and guiding (teacher, sports, lighthouse, energy source), relaxing and peaceful (worshipping, lightning rod, nature, sea, sun), flexibility and elegance (swan, flamingo, butterfly, child) themes.

*Worshipping: I can compare yoga to worshipping because I think it is a spiritual and meditative action. I feel that I am getting closer to God. (P1)*.

*Sports: The human body is an excellent machine. Yoga is a sport that makes this machine work most perfectly for many years. All organs support each other physically and chemically. The brain governs it (P2).*

*Lightning rod: I would compare it to a lightning rod. Because I think that yoga grounds the tense energy accumulated on the body, just as the lightning rod provides grounding without damaging the building where the lightning strikes (P9).*

*Nature: The only thing which I can compare yoga to is nature's own cycle. Because while we are practising yoga as a part of nature, we begin to stretch at first, and move slowly with breathing exercises. Then we get caught up in the flow of yoga, just like the flow of nature. Finally, we finish the action of yoga as nothing in nature is infinite. However, since there is no end in nature, yoga is also completely unfinished, and we turn into another type, mood and energy frequency (P3).*

*Sun: I compare yoga to the sun because it warms me inside (P4).*

*Sea: I think of yoga practice as the peace/infinite happiness that the still/calm blue sea gives me (P5). I compare yoga to the deep blue sea with its visible bottom, creating waves towards the golden sand, containing cleanliness, clarity and peace in it (P10).*

*Lighthouse: Yoga prevents you from getting lost by allowing you to find your direction like a lighthouse (P6).*

*Energy source: I would say yoga is an energy source of life because I would change my whole life according to yoga. This has been my belief. (P8).*

*Flamingo: I compare practising yoga to a flamingo standing on one leg. Flamingos regulate their body temperature by being able to stand on one leg for a long time (P11).*

*Child: I compare yoga to a child. Because they can make every move with their flexible body (P13).*

*Butterfly: I would compare yoga to flying like a butterfly over the fragrant coloured flowers in a lush environment on the river bank. Because that's how I feel when I practise yoga (P15).*

*Teacher: I compare yoga to a teacher. It teaches us our spiritual and physical body. It teaches the spiritual depths of the body to turn back to the essence (P16).*

*Swan: I feel like a swan while practising yoga, the climax of emotions, elegant movements, movements as if you are evolving into nature (P18).*

## Discussion

With this study, the experiences of individuals who have been practicing yoga for a long time were revealed. The benefit perceived by adults who practiced yoga for a long time was demonstrated in terms of physical, mental, and social aspects by qualitative analysis method. The findings obtained from the study showed that at the end of the yoga practice, which was applied two sessions per week for two years, the individuals had reductions in pain and flexibility, relaxation in the spiritual field, higher sleep quality, positive personality traits, self-esteem, and positive emotion, and they were effectively able to cope with anxiety and stress.

Because the study was qualitative the situations experienced by the individuals, their reactions to events and their beliefs, attitudes and behaviours were evaluated in a more realistic, systematic and detailed way. The original aspect of this study is that it is both a qualitative study and that the participants became aware of the positive effects of yoga in daily life and adopted the habit of regularly practicing yoga. One of the conclusions that can be obtained from this study is that starting yoga early and continuing it for a long time will relieve physical difficulties. Thus, individuals can be encouraged to participate in daily life and their quality of life can be improved by increasing their yoga physical fitness and physical activity levels.

For the participants participating in this study, yoga has different meanings, such as exercise, healthy life, worshipping, calmness, harmony, awareness, and balance. The origin of the word "yoga" in the literature is etymologically based on the Sanskrit words "joug" and "yuj (g)" which mean "connection" and "unity". As a thought, yoga means that all different aspects that make up the human being are combined with it in a balanced way. In this context, yoga is also expressed as a “life system’ that explains the basic principles of life and the “truth” in its entirety without breaking it down [17]. In a study on yoga, the meaning of yoga was expressed as body awareness, energy and physical, mental and emotional integrity caused by physical posture, breathing work and relaxation [18]. In another study, participants interpreted yoga as regeneration, moral force and, awareness of the body, a finding similar to our study [19].

A study by Kuru, Alici and colleagues [20], similarly stated that for participants, yoga meant personal transformation. It is known in the literature that yoga has positive contributions to both healthy people and those with health problems in the physical, social, and spiritual aspects. Similar to the results of the study, other studies have shown that yoga improves mood [21], reduces stress [22], increases well-being [23] and improves the quality of life [3, 24].

In this study, metaphor terms related to practicing yoga were usually collected within the framework of the supporting, guiding, relaxing, peaceful, flexibility and elegance themes. The study showed that the metaphors for practising yoga were in harmony with the literature. It is generally accepted in the literature that yoga creates physical and psychological benefits, and it is generally reported that it can be used for strengthening muscles, reducing stress, and maintaining the physical and psychological well-being of the person, and relieving the ailments such as fatigue, pain, difficulty in concentration and loss of balance [25]. Yoga is also a lifestyle activity, and lifestyle contributes to the continuity of the ontological sense of security. We, as human beings, need ontological security due to our concern about the continuation of our existence. We manage this anxiety through daily routine, social interactions, and lifestyle choices [26]. Yoga practice aims to improve or maintain physical fitness and health [8]. The perception of yoga as a part of physical culture has been attributed to its stress-reducing and relaxing properties depending on anatomical and physiological assumptions [27]. Physical activity stimulates several brain chemicals that make you feel happier, much more relaxed, and less anxious. Scientific studies show that physical activity performed through yoga can decrease depression. The results of this study revealed positive improvements with yoga from the aspects of feeling good and positive emotions, more positive perception of one's body image, physical self-esteem, and self-pride. Moreover, as in similar studies, it was seen in this study that yoga decreased stress, improved stress-coping power, and positively affected the quality and duration of sleep among those having sleep disorders [9–11, 27, 28].

In this study, female participants were in the majority as in other studies [28, 29]. In this study, only two of the eighteen participants were male. When yoga studies are examined, it is seen that yoga is a field where women are predominant. The reasons for this situation are the effects of certain gender roles and stereotypes about women and men. It is accepted that women are more open to innovation, spiritual practices, and accepting their shortcomings. These differences that are accepted as existing between men and women are based on gender roles. According to the results of the research, it was revealed that not practicing yoga is a result of prejudice in men and that they are worried that they will experience social prejudice. The data obtained from studies showing the socio-demographic characteristics of yoga practitioners in Turkey are similar to the results of this study [7, 29, 30].

## Limitations of study

The results of this study were limited to people who practice yoga in a particular institution. In addition, due to the nature of the qualitative study, it only covers the answers of eighteen participants.

## Conclusions

The study has enabled us to deeply understand what yoga is and how it affects people. It has been found that problems such as pain and anxiety that individuals face in their daily life can be more easily solved with regular yoga practice. Yoga is a slow and elegant activity suitable for all participants that can improve the life energy of the participants. Practicing yoga has brought about positive developments in the subjects, such as feeling good and positive, experiencing inner comfort and peace, feeling stronger in their world of belief, perceiving their own body images more positively, valuing themselves physically and being proud of themselves. It is recommended that the effects of yoga be revealed by qualitative research in different populations. It is recommended that yoga be started at an early age and done regularly as it provides easy resolution of significant problems such as pain, anxiety, sleeping, stress, that negative affect our daily life.

## Data Availability

The data and materials of the current study are available from the corresponding author on reasonable request.
